# Insanity and Its Treatment

**Published:** 1871-05

**Authors:** 


Insanity and its Treatment: Lectures on the Treatment, Medical and Legal, of Insane Patients. By G. Fielding Blandford, M.D., Oxon., with a Summary of the Laws in Force in the United States on the Confinement of the Insane, by Isaac Ray, M.D. Philadelphia: Henry C. Lea, publisher.

This is a neatly published volume of 470 octavo pages. It would form a very useful work in any practitioner's library, and its contents are of great value to those specially interested in the nature and treatment of the insane.

